# Regional Tongue Deformations During Chewing and Drinking in the Pig

**DOI:** 10.1093/iob/obab012

**Published:** 2021-04-22

**Authors:** Rachel A Olson, Stéphane J Montuelle, Hannah Curtis, Susan H Williams

**Affiliations:** 1 Department of Biological Sciences, Ohio University, Athens, OH 45701, USA; 2 Department of Biomedical Sciences, Ohio University Heritage College of Osteopathic Medicine, Warrensville Heights, OH 44122, USA; 3 Department of Biomedical Sciences, Ohio University Heritage College of Osteopathic Medicine, Athens, OH 45701, USA

## Abstract

As a muscular hydrostat, the tongue undergoes complex deformations during most oral behaviors, including chewing and drinking. During thesebehaviors, deformations occur in concert with tongue and jaw movements to position and transport the bolus. Moreover, the various parts of the tongue may move and deform at similar timepoints relative to the gape cycle or they may occur at different timepoints, indicating regional biomechanical and functional variation. The goal of this study is to quantify tongue deformations during chewing and drinking in pigs by characterizing intrinsic changes in tongue dimensions (i.e., length and width) across multiple regions simultaneously. Tongue deformations are generally larger during chewing cycles compared to drinking cycles. Chewing and drinking also differ in the timing, relative to the gape cycle, of regional length and width, but not total length, deformations. This demonstrates functional differences in the temporal dynamics of localized shape changes, whereas the global properties of jaw–tongue coordination are maintained. Finally, differences in the trade-off between length and width deformations demonstrate that the properties of a muscular hydrostat are observed at the whole tongue level, but biomechanical variation (e.g., changes in movements and deformations) at the regional level exists. This study provides new critical insights into the regional contributions to tongue deformations as a basis for future work on multidimensional shape changes in soft tissues.

## French

En tant qu'hydrostat musculaire, la langue subit des déformations complexes pendant la plupart des comportements oraux, en particulier au cours de la mastication et de l’ingestion de liquide. Au cours de ces comportements, les déformations se produisent de concert avec les mouvements de la langue et des mâchoires pour positionner et transporter le bolus. De plus, les différentes parties de la langue peuvent se déplacer et se déformer à des moments similaires ou différents par rapport au cycle d’ouverture de la bouche, indiquant une variation biomécanique et fonctionnelle régionale de la langue. L'objectif de cette étude est de quantifier les déformations de la langue pendant la mastication et l'ingestion d’eau chez le porc en caractérisant les changements intrinsèques des dimensions de la langue (i.e., longueur et largeur) des différentes régions de la langue simultanément. Les déformations de la langue sont généralement plus importantes pendant les cycles de mastication que pendant les cycles d’ingestion d’eau. La mastication et l'ingestion d’eau diffèrent également dans le timing (par rapport au cycle d'ouverture de la bouche) des déformations régionales de la langue en longueur et en largeur, mais pas en longueur totale. Cela démontre des différences fonctionnelles dans la dynamique temporelle des changements localisés de la forme de la langue alors que les propriétés globales de la coordination mâchoire-langue sont maintenues. Enfin, les différences dans le compromis mettant en jeu les déformations en longueur et en largeur démontrent que les propriétés d'un hydrostat musculaire sont observées au niveau de la langue entière, mais qu'il existe une variation biomécanique (par exemple, des changements dans les mouvements et les déformations) au niveau régional. Cette étude fournit de nouvelles informations essentielles sur les contributions régionales des déformations de la langue, qui serviront de base aux travaux futurs sur les changements multidimensionnels de forme dans les tissus mous.

## Introduction

Like other muscular hydrostats, the muscular tongue of vertebrates has a complex arrangement of muscles that allows it to deform in myriad ways while maintaining a constant volume (Kier and Smith 1985; Smith and Kier 1989). The muscles forming the tongue consist of interdigitating intrinsic and extrinsic muscle fiber bundles. While some of the external fiber bundles originate from the hyoid apparatus, the tongue lacks an internal bony support system that would otherwise confer rigidity. This frees the tongue from the functional constraints of a typical muscle with bony origins and insertions, allowing it to deform and move in 3 dimensions as it performs a variety of oral functions such as feeding and drinking.

Although static interpretations of deformations can be made from the muscle anatomy of muscular hydrostats (Kier and Smith 1985; Smith and Kier 1989), the dynamic nature of these deformations can be difficult to predict, particularly at a regional level. This is particularly true for mammalian tongues because, unlike many other muscular hydrostats that exhibit a fairly constant anatomic arrangement of muscles throughout the structure, the body of the tongue is heterogeneous in its muscle anatomy (Napadow et al. 1999a; Sokoloff and Burkholder 2012). This heterogeneity is due, in part, to the manner and location in which the extrinsic muscles enter the tongue and interdigitate with the intrinsic muscles to contribute to its structure and function. For example, the styloglossus muscle joins the posterior region of the tongue to interdigitate on its lateral aspect with the intrinsic longitudinal muscles, whereas the genioglossus enters the body of the tongue in a fan-like pattern with a mostly vertical orientation (Sokoloff and Burkholder 2012). Recent evidence also suggests that the suprahyoid muscles may also play a role in tongue deformations through a hydraulic linkage mechanism involving the hyoid and oral floor (Orsbon et al. 2020).

Relationships between tongue form and function have been studied with magnetic resonance imaging (MRI) studies of human speech (e.g., Napadow et al. 1999b; Stone et al. 2010) and swallowing (e.g., Napadow et al. 1999a). In these studies, specific deformations of tongue shape have been correlated with the surrounding muscle fiber orientation. Most MRI studies provide a singular instantaneous view of deformations during highly controlled behaviors, and they do not always consider movements of attached structures, such as the jaw or hyoid. However, other oral behaviors such as chewing and drinking rely on tongue movements and deformations in coordination with the jaw to manipulate a bolus. Thus, MRI imaging is limited in its capacity to characterize dynamic shape changes at the cycle or sequence level and relate these to gape cycle dynamics.

More invasive sonomicrometry studies in the pig demonstrate regional differences in the magnitude and timing of tongue deformations relative to opening and closing of the jaw during feeding and drinking (Liu et al. 2007, 2009). During chewing, tongue width increases during occlusion through jaw opening, whereas tongue length and posterior tongue dorsoventral thickness generally increase during jaw opening into jaw closing. Functionally, these sonomicrometry studies indicate that the tongue lengthens and thickens during the food handling portions of the cycle (jaw closing and opening) and widens during the occlusal phase when the upper and lower postcanine teeth and food are in contact. These deformations function to keep food in the mouth during jaw opening and push food against the hard palate during the occlusal period (Liu et al. 2009). Results from this study also suggest that constant volume may be preserved across the whole structure of the tongue instead of at a regional level (Liu et al. 2009). While the benefit of this work is a more biologically relevant interpretation of tongue biomechanics in the context of normal oral function, it does not fully capture the potential complexity and variability that may occur on a finer scale (i.e., across the different parts of the tongue) during feeding. The anatomical heterogeneity observed in mammalian tongues suggests there is potential for biomechanical differences in different regions of this structure, and thus differences in function. By describing intrinsic dynamic deformations of the mammalian tongue on a regional scale, we can understand how its anatomical heterogeneity contributes to regional shape change during oral behaviors.

Here, we investigate fine-scale regional tongue deformations to characterize and compare fundamental aspects of the tongue’s biomechanical heterogeneity during 2 rhythmic oral behaviors, chewing (i.e., mastication) and drinking, in the pig (*Sus scrofa*, Linnaeus 1758). First, we describe overall patterns of tongue deformation during chewing and drinking gape cycles. Next, we compare the magnitude of these changes to test a series of hypotheses about tongue deformations during chewing and drinking. We hypothesize that during chewing tongue length and width will deform more than during drinking. This hypothesis is based on our previous work demonstrating that tongue protraction–retraction movements are more pronounced during chewing (Olson et al. 2021) and assume that length deformations, and corresponding inverse changes in width, are in part contributing to these positional changes of the tongue. For example, as the tongue is protracted, anteroposterior (AP) tongue lengthening and mediolateral (ML) narrowing will also occur. However, we expect the timing of these deformations to be generally similar between behaviors, reflecting the fundamental constraints of jaw–tongue coordination that not only protects the tongue but also functions to transport food or liquid into and/or within the oral cavity. Additionally, we qualitatively assess whether dimensional changes in length and width at a regional level are consistent with the muscular hydrostat model, which predicts that expansion in one dimension is compensated for by compression in another dimension. Lack of an observable pattern would not necessarily suggests that the regions are not behaving according to this model because compensatory dorsoventral dimension might be occurring as well, as suggested by Liu et al. (2009) for the most caudal portion of the anterior two-thirds of the tongue (i.e., tongue base). Nevertheless, if tradeoffs are observed between regional lengths and widths, it would suggest that dorsoventral changes may actually not play a large role in maintaining the muscular hydrostat properties of the tongue.

## Materials and methods

### Data collection

**Fig. 1:**
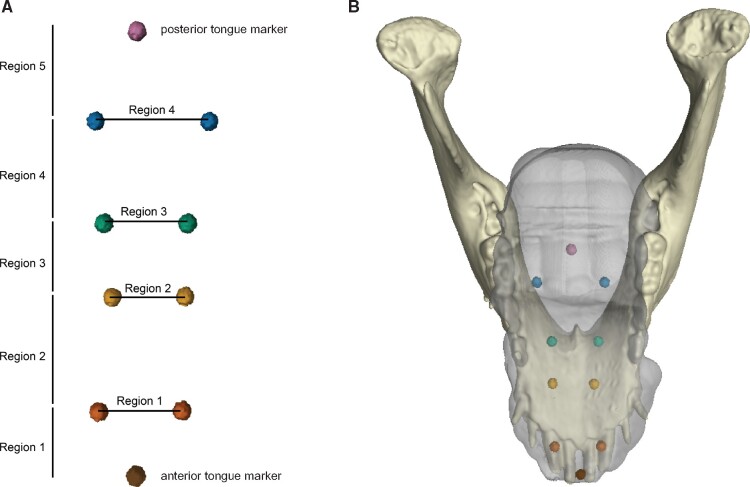
(**A**) Schematic of tongue bead distribution indicating markers used to determine normalized lengths and widths and regional deformations. (**B**) Locations of the tongue markers relative to the jaw when the tongue is at rest within the oral cavity.

Regional deformations of the tongue were quantified relative to the gape cycle during chewing and drinking in 2 3-month-old Hampshire-cross pigs using marker-based X-ray Reconstruction of Moving Morphology (XROMM) with additional soft tissue markers in the tongue (Brainerd et al. 2010). Following our previously published XROMM protocols (e.g., Montuelle et al. 2019, 2020), a minimum of 5 1.6 mm tantalum markers (Bal-tec, Los Angeles, CA, USA) were aseptically implanted under isoflurane anesthesia into both the skull and jaw of each animal. Using a sterile hypodermic needle, an additional 17 markers, of which 10 were analyzed for this study (Fig. 1), were implanted into the body of the tongue. The anterior-most marker was positioned at the tip of the tongue, ∼2 mm posterior to the anterior-most point, and the posterior-most marker was inserted immediately anterior to the circumvallate papillae. The marker pairs were approximately equally spaced between these 2 markers to create 5 regions. Markers were not positioned in a way to measure muscle length changes, as in traditional fluoromicrometry, because they span multiple muscles with different orientations (see Camp et al. 2016). Final resting position of the markers from the computed tomography (CT) scan is reflected in Supplementary Table S1. During the week-long recovery period, animals were CT scanned at The Ohio State University College of Veterinary Medicine (Columbus, OH, USA) on a GE Lightspeed Ultra CT scanner while under isoflurane anesthesia. These scans were used to produce the XROMM animations.

For an experimental trial, animals fed on 2× 2 × 1 cm cubes of apple or drank apple juice from a bowl while being recorded at 250 fps by 2 synchronized Oqus 310 cameras (Qualisys, Göteborg, Sweden) mounted on the output ports of 2 synchronized OEC-9000 fluoroscopes (General Electrics, Boston, MA, USA). Radiation technique averaged 100 kVp and 4.3 mA across trials. Fluoroscopy videos and a separate webcam recording at 30 fps were synchronized and saved in Qualisys Track Manager motion capture software. Trials of each behavior were recorded over the course of a week. Prior to each recording session, perforated metal sheets (part number 9255T641, McMaster-Carr, Robinson, NJ) and a custom Lego^®^ calibration cube were imaged in each fluoroscopy view to undistort and calibrate the videos, respectively, in XMALab (Knörlein et al. 2016). At the termination of the study, animals were euthanized with an intravenous injection of sodium pentobarbital while under isoflurane anesthesia. A post-mortem CT scan was performed at Holzer Clinic (Athens, OH) on a Philips Brilliance 64 scanner for the precision study (see below). All procedures involving live animals were approved by the Ohio University Institutional Animal Care and Use Committee (protocol #12-U-009).

### Video processing and XROMM animations

Fluoroscopy videos were processed according to the XROMM workflow in XMALab to (1) track 2D points of each marker in the undistorted and calibrated field of view, (2) calculate their respective 3D coordinates, and (3) quantify and filter (low-pass Butterworth, 25 Hz cut-off frequency) rigid body transformations of the skull and the jaw (Brainerd et al. 2010; Knörlein et al. 2016). The marker-tracking precision (i.e., mean standard deviation [SD] of markers) was 0.85 ± 0.429 for intraosseous markers and 0.44 ± 0.343 for tongue markers for Pig 20 and 0.68 ± 0.445 for intraosseous markers and 0.64 ± 0.457 for tongue markers for Pig 21. Meshes of bones and all tantalum markers from the CT scans were created in VGSTUDIO MAX version 3.3 (Volume Graphics GmbH), and animations of the reconstructed CT models were created in MAYA (Autodesk Inc., San Rafael, CA) using the 3D coordinate data. The 3D position of the centroids of the tongue markers at rest was calculated in MAYA from the bead mesh and used for normalization (see below). For animations, tongue markers were animated as locators using their filtered 3D locations from XMALab. These animations were then used to visualize, quantify, and export jaw and tongue movements relative to the skull.

Five AP and 4 ML tongue regions, with their respective regional lengths and widths, were defined based on pairs of tongue markers (Fig. 1A). Changes in regional lengths and widths were measured throughout the gape cycle. Lengths were calculated from the 3D distance between 2 consecutive midpoints of right-left marker pairs, or a single marker in the midline for the anterior- and posterior-most tongue markers. Regional widths were calculated from the 3D distance between right and left marker pairs. In order to account for variation in bead placement and minor size differences between individuals, lengths, and widths were normalized to the resting distance between marker pairs extracted from the corresponding *in vivo* CT scan when the tongue was positioned in a relaxed neutral position inside the oral cavity (Fig. 1B; Supplementary Table S1). Changes in total tongue length were calculated as the sum of each of the regional deformations in order to account for off-axis shape changes.

### Data analysis

The magnitude and timing of regional tongue deformations were determined relative to the gape cycle and gape cycle phases. Gape cycle dynamics were determined by the direction and acceleration of jaw rotation about the *z*-axis (i.e., Rz, jaw pitch) of a 3-axis joint coordinate system following Brainerd et al. (2010). This was automated in FeedCycle, a custom MATLAB script (Dr Brad Chadwell, Idaho College of Osteopathic Medicine) that first determines the start, end, and transition between jaw opening and closing of each gape cycle. The second derivative of Rz position change (acceleration) then identifies the transition points between the slow and fast phases of jaw opening and closing. Chewing cycles consistently had 4 phases (i.e., fast close [FC], slow close [SC], slow open [SO], and fast open [FO]), whereas drinking had 3 phases (i.e., closing [C], open 1 [O1], and open 2 [O2]). Phases were compared based on directionality (i.e., opening or closing) and acceleration of Rz, such that FC of chewing was comparable to C of drinking and SO and FO of chewing were compared to O1 and O2 of drinking, respectively (see Olson et al. 2021).

**Fig. 2:**
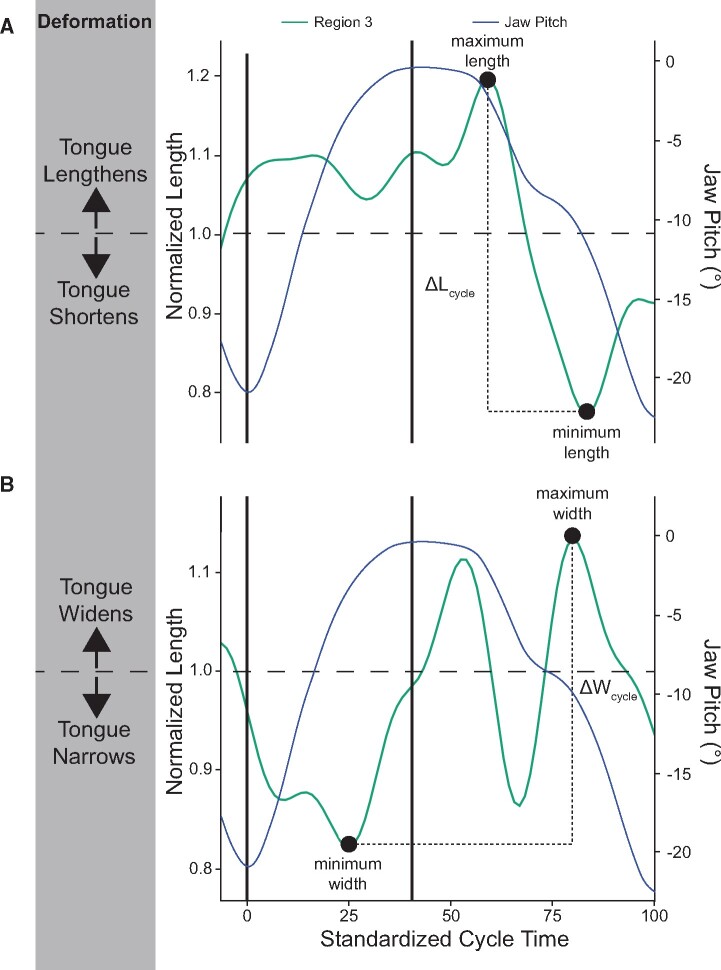
Representative graph of normalized length (**A**) and width (**B**) changes from resting length and width, respectively, in R3 during a single chewing cycle. The corresponding trace of Rz (blue) is also shown. Values >1 indicate an increase from resting position and values <1 indicate a decrease from resting position. ΔL_cycle_ and ΔW_cycle_ represent the magnitude of length and width changes during the cycle.

For each cycle, FeedCycle identified the maximum and minimum normalized total and regional tongue lengths and regional widths (Fig. 2). To determine the magnitude of deformation of each region for each cycle, ΔL_cycle_ and ΔW_cycle_ were calculated from the corresponding regional cycle maximum and minimum lengths and width, respectively (Fig. 2A and B). The timing of each of these maximum and minimum values relative to the gape cycle phases was also extracted. Timing parameters were adjusted to standardized cycle time, reflected as a percentage of cycle duration.

For the final dataset used for statistical analysis, we discarded all nonchewing and nondrinking cycles from each sequence as well as any cycle containing a swallow. This resulted in 102 chewing cycles (47 for Pig 20 and 55 for Pig 21) and 90 drinking cycles (40 for Pig 20 and 50 for Pig 21). All statistical analyses were performed in R version 3.6.1 (R Core Team 2019). The lme (*nlme: Linear and Nonlinear Mixed Effects Models*. R package version 3.1-143; Pinheiro et al. 2019) and *Estimated Marginal Means, aka Least-Squares Means* (R package version 1.5.1; Lenth 2019) functions were used to run linear mixed effects models with repeated measures to compare tongue deformation magnitudes, with behavior (chew and drink) as a fixed effect and individual as the random effect. CircStats (*CircStats*. R package version 0.2-6; Lund and Agostinelli 2019) was used to calculate circular means (i.e., mean of timing parameter adjusted to standardized cycle time) for timing parameters and variance used in figures. For timing parameter models, we followed the methods of Cremers and Klugkist (2018) to conduct Bayesian circular mixed effects models with repeated measures, with behavior (chew, drink) as a fixed factor and individual as the random factor. For this, we used the bpnme function (10,000 iterations, 2000 burn-in, 101 seed, n.lag = 3) from the package *Bayesian Projected Normal Regression Models for Circular Data* (*bpnreg*; R package version 1.0.3; Cremers 2019) (see Olson et al. 2021). Traditional mixed effects models would view timepoints at 5% and 95% of standard cycle duration as being located 90% apart, when they are only 10% different. This has an important functional implication in that traditional analyses would result in an average of these timepoints at 50% of the cycle, around minimum gape, when in reality both occur very close to maximum gape. A Bayesian approach allows for the calculation of mixed effects models in circular space (see details in Cremers and Klugkist 2018; see e.g., Olson et al. 2021). Bpnreg produces the posterior mean, posterior SD, and the 95% highest posterior density (HPD) interval. HPDs are reported as the directionally dependent start position (as percentage of cycle duration) to end position. When HPDs are nonoverlapping, there is a difference between behaviors. When HPDs are overlapping, the null hypothesis of no difference between behaviors cannot be rejected.

Finally, we assess whether there are qualitatively observable patterns of compensatory changes in AP regional deformations and corresponding ML deformations to suggest that the properties of a muscular hydrostat are preserved at regional levels for both behaviors. For example, we would expect high AP deformation is associated with low ML deformation and low AP deformation with high ML deformation. Note that this assessment is exploratory in nature given that we do not assess the changes over time (e.g., through a gape cycle) and we do not assess changes in the dorsoventral dimension.

## Results

### General patterns of tongue deformations during chewing and drinking

#### Length

**Fig. 3:**
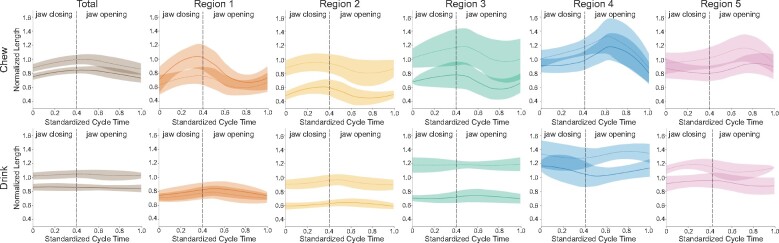
Means and 95% confidence intervals of normalized total and regional lengths during chewing (top) and drinking (bottom). Greater length changes are observed during chewing for all regions. In each plot, Individual 20 is indicated by solid lines and Individual 21 by dashed lines. Individual gape cycles are standardized to the same length, with the initiation of jaw closing occurring at 0. The mean time of minimum gape is indicated by the vertical dashed line. Sample size: 102 chewing cycles (47 for Pig 20; 55 for Pig 21) and 90 drinking cycles (40 for Pig 20; 50 for Pig 21).

Total tongue length changes little within chewing and drinking cycles, never lengthening >1.1× resting length. Compared to drinking, chewing involves overall larger total tongue deformations, and even more pronounced regional deformations (Fig. 3). During chewing, length increases through jaw closing, peaks slightly before or after minimum gape, and subsequently decreases through much of jaw opening until it begins to increase at the end of opening prior to the start of the next cycle. The timing of these deformations is most noticeable for the anterior-most regions of the tongue (R1–R3). More posteriorly (i.e., for R4 and R5), lengthening is more subtle through jaw closing, or even shortening (R5, Pig 20), and maximum length occurs around midway through opening after which these regions then shorten. Thus, the overall pattern of lengthening and shortening direction is preserved, but their timing is offset regionally from anterior to posterior. During drinking, comparatively small deformations occur for all regions, and there is not as clear of a pattern as in chewing.

#### Width

**Fig. 4:**
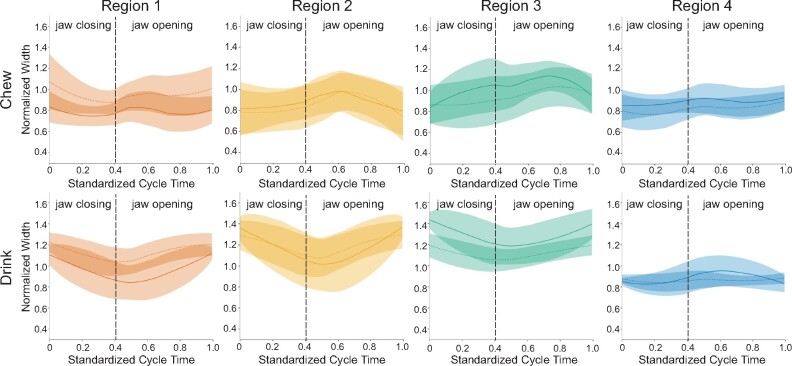
Means and 95% confidence intervals of normalized regional tongue widths during chewing (top) and drinking (bottom). Similar amounts of deformation occur during both behaviors. In each plot, Individual 20 is indicated by solid lines and Individual 21 by dashed lines. Individual gape cycles are standardized to the same length, with the initiation of jaw closing occurring at 0. The mean time of minimum gape is indicated by the vertical dashed line. Sample size: 102 chewing cycles (47 for Pig 20; 55 for Pig 21) and 90 drinking cycles (40 for Pig 20; 50 for Pig 21).

The magnitude of changes in normalized regional widths throughout the gape cycle is similar during chewing and drinking, staying between 0.5× and 1.5× resting length across all regions, whereas behavior-specific patterns in timing are evident (Fig. 4). For both behaviors, R4 undergoes the least amount of total ML deformation through the cycle. During chewing, the normalized width of R1 is bimodal, with one peak at the start of the gape cycle around maximum gape and a second peak after minimum gape. R2, R3, and R4 peak once during jaw opening. In contrast, during drinking, the timing of maximum width occurs around maximum gape for R1–R3 and just after minimum gape for R4.

### Magnitude of normalized deformations

#### Length

As hypothesized, the magnitude of the total and regional changes in length (ΔL_cycle_) are significantly larger during chewing than drinking due to statistically higher maximum values (except R2) and lower minimum values (Table 1). For both behaviors, all AP deformation occurs within 0.5–1.5× resting total or regional length, but the more posterior regions (R3–R5) undergo greater deformation than the anterior regions (Table 1).

**Table 1:** Model results and summary statistics of length parameters

Tongue Region	ΔL_cycle_			Maximum normalized length			Minimum normalized length		
	Mean ± SD			Mean ± SD			Mean ± SD		
	Chew	Drink	Model	Chew	Drink	Model	Chew	Drink	Model
Total	0.16 ± 0.034	0.07 ± 0.016	SE = 0.0035 T_2,192_ = 26.2 *P* < 0.0001	0.96 ± 0.842	0.99 ± 0.943	SE = 0.0038 T_2,192_ = −7.4 *P* < 0.0001	0.80 ± 0.627	0.92 ± 0.093	SE = 0.0038 T_2,192_ = −31.9 *P* < 0.0001
R1	0.37 ± 0.118	0.14 ± 0.022	SE = 0.0101 T_2,192_ = 22.4 *P* < 0.0001	0.95 ± 0.153	0.84 ± 0.043	SE = 0.0112 T_2,192_ = 8.8 *P* < 0.0001	0.58 ± 0.055	0.71 ± 0.038	SE = 0.0051 T_2,192_ = −25.2 *P* < 0.0001
R2	0.28 ± 0.063	0.12 ± 0.028	SE = 0.0061 T_2,192_ = 26.6 *P* < 0.0001	0.85 ± 0.200	0.86 ± 0.166	SE = 0.0063 T_2,192_ = −0.4 *P* = 0.679	0.57 ± 0.163	0.73 ± 0.149	SE = 0.0050 T_2,192_ = −33.0 *P* < 0.0001
R3	0.40 ± 0.123	0.12 ± 0.033	SE = 0.0125 T_2,192_ = 22.6 *P* < 0.0001	1.08 ± 0.255	1.03 ± 0.220	SE = 0.0113 T_2,192_ = 4.8 *P* < 0.0001	0.68 ± 0.166	0.91 ± 0.236	SE = 0.0091 T_2,192_ = −25.1 *P* < 0.0001
R4	0.48 ± 0.097	0.20 ± 0.052	SE = 0.0114 T_2,192_ = 24.6 *P* < 0.0001	1.36 ± 0.119	1.31 ± 0.107	SE = 0.0106 T_2,192_ = 5.5 *P* < 0.0001	0.88 ± 0.063	1.11 ± 0.139	SE = 0.0106 T_2,192_ = −21.0 *P* < 0.0001
R5	0.34 ± 0.095	0.19 ± 0.047	SE = 0.0100 T_2,192_ = 15.7 *P* < 0.0001	1.15 ± 0.130	1.12 ± 0.102	SE = 0.0061 T_2,192_ = 5.3 *P* < 0.0001	0.81 ± 0.073	0.94 ± 0.110	SE = 0.0075 T_2,192_ = −16.7 *P* < 0.0001

SE, standard error.

#### Width

The magnitude of width change (ΔW_cycle_) was also significantly higher for chewing than for drinking for all tongue regions, and the ranges of width deformations also all occur within 0.5–1.5× resting length (Table 2). In contrast to chewing, drinking has higher regional maximum and minimum widths, except for R4, which does not differ between behaviors (Table 2). R4 also has the smallest range of width deformations because it rarely exceeds its resting width.

**Table 2:** Model results and summary statistics of width parameters

Tongue Region	ΔW_cycle_			Maximum normalized width			Minimum normalized width		
	Mean ± SD			Mean ± SD			Mean ± SD		
	Chew	Drink	Model	Chew	Drink	Model	Chew	Drink	Model
R1	0.32 ± 0.060	0.28 ± 0.045	SE = 0.0078 T_2,192_ = 4.9 *P* < 0.0001	1.04 ± 0.112	1.17 ± 0.070	SE = 0.0064 T_2,192_ = −19.8 *P* < 0.0001	0.73 ± 0.081	0.89 ± 0.102	SE = 0.0050 T_2,192_ = −32.7 *P* < 0.0001
R2	0.39 ± 0.061	0.37 ± 0.075	SE = 0.0079 T_2,192_ = 2.0 *P* = 0.0458	1.09 ± 0.054	1.34 ± 0.031	SE = 0.0056 T_2,192_ = −44.1 *P* < 0.0001	0.70 ± 0.049	0.96 ± 0.058	SE = 0.0073 T_2,192_ = −36.2 *P* < 0.0001
R3	0.37 ± 0.093	0.25 ± 0.070	SE = 0.0099 T_2,192_ = 12.2 *P* < 0.0001	1.17 ± 0.093	1.32 ± 0.111	SE = 0.0090 T_2,192_ = −16.5 *P* < 0.0001	0.80 ± 0.079	1.07 ± 0.070	SE = 0.0097 T_2,192_ = −27.7 *P* < 0.0001
R4	0.22 ± 0.055	0.18 ± 0.068	SE = 0.0080 T_2,192_ = 5.1 *P* < 0.0001	0.97 ± 0.068	0.96 ± 0.063	SE = 0.0063 T_2,192_ = 0.04 *P* = 0.967	0.75 ± 0.060	0.79 ± 0.021	SE = 0.0060 T_2,192_ = −6.8 *P* < 0.0001

SE, standard error.

### Timing of tongue deformations relative to gape cycle phases

#### Length

**Fig. 5:**
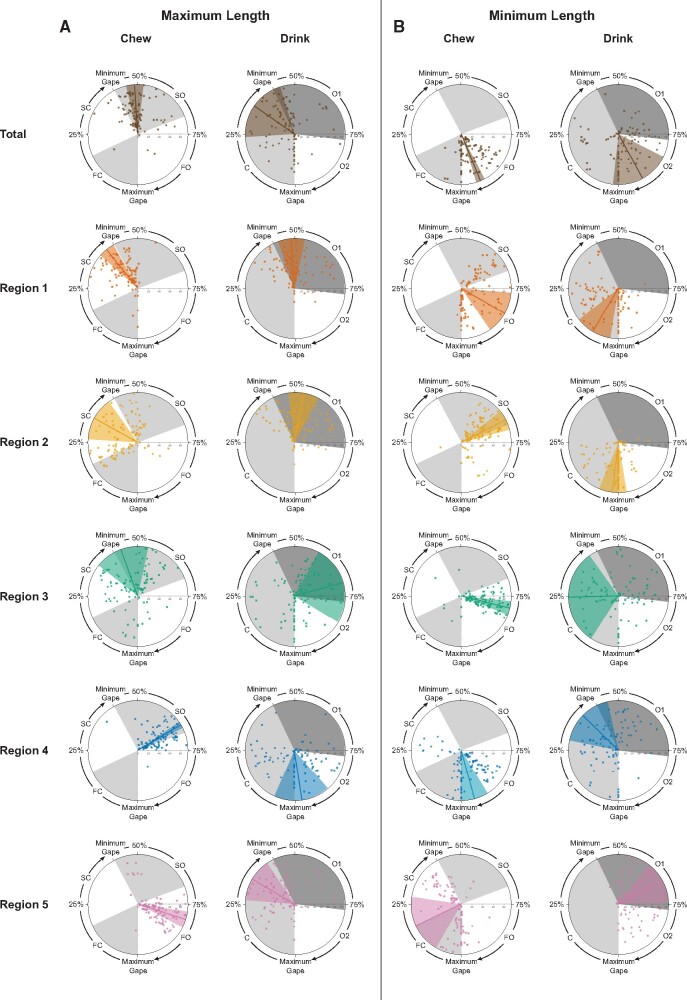
The timing of maximum and minimum length deformations relative to the gape cycle and mean phase durations were not always similar between behaviors. In each plot, the timing of maximum (left) or minimum (right) length deformation for the total tongue and each tongue region is expressed as a percent of total cycle duration and shown relative to wedges representing relative mean phase durations (alternating gray and white) during chewing and drinking (see Supplementary Figure S1). Lines indicate mean values and wedges show the corresponding variance. Individual 20 is indicated by circles and Individual 21 by squares. The location on the radius at 75% of the gape cycle indicates cycle number in the sequence with more centrifugal points corresponding to cycles later in the sequence.

We hypothesized that the timing of maximum and minimum normalized tongue total and regional lengths would occur at similar times in the gape cycle during chewing and drinking because of the overall constraints of jaw–tongue coordination. Results for maximum total length are consistent with this hypothesis. As shown in Fig. 5A, maximum total length occurs near minimum gape during both behaviors. The overlapping HPD intervals of 43.1–51.2 (chew) and 5.0–47.8 (drink) indicate no significant differences between the behaviors (Table 3). No difference was observed between behaviors in the maximum length of R2 or R3 as well (Table 3), even though the means did not occur in the same intracycle phase (Fig. 5A). In contrast, the nonoverlapping HPD values for R1, R4, and R5 indicate differences in the timing of maximum length during chewing and drinking for these regions (Table 3). Maximum R1 length occurs just prior to minimum gape for chewing but shortly after for drinking (Fig. 5A). For R4, maximum length during chewing occurs around the SO–FO transition, whereas for drinking it occurs around maximum gape. Finally, R5 maximum length occurs during FO for chewing whereas during drinking it occurs late in the closing phase (Fig. 5A).

**Table 3:** Results of the circular mixed effects model of the timing of maximum and minimum AP (length) and ML (width) deformation, as a percentage of standardized cycle time

Tongue Region	Maximum length		Minimum length		Maximum width		Minimum width	
	Chew	Drink	Chew	Drink	Chew	Drink	Chew	Drink
Total	**48.0 ± 5.71 (43.1–51.2)**	**32.2 ± 12.70 (5.0–47.8)**	**93.9 ± 1.56 (92.1–95.7)**	**91.5 ± 5.34 (81.6–99.2)**	NA	NA	NA	NA
R1	39.8 ± 0.89 (38.2**–**41.4)	48.4 ± 1.38 (45.9**–**50.9)	83.8 ± 8.32 (63.9**–**97.2)	9.1 ± 7.23 (1.1**–**28.5)	**78.6 ± 14.61 (53.0–98.3)**	**97.4 ± 5.36 (90.6–1.8)**	79.5 ± 9.50 (72.2**–**21.8)	48.7 ± 7.04 (35.1**–**64.9)
R2	**33.3 ± 6.97 (19.2–50.4)**	**53.2 ± 5.38 (42.9–65.2)**	66.4 ± 1.50 (64.0**–**68.7)	2.1 ± 1.66 (99.2**–**4.9)	64.3 ± 1.49 (62.0**–**66.5)	0.4 ± 1.61 (98.0**–**2.8)	99.7 ± 6.35 (85.2**–**12.5)	52.3 ± 4.31 (43.3**–**62.0)
R3	**43.8 ± 6.93 (27.2–52.1)**	**70.0 ± 12.24 (51.7–95.1)**	**78.6 ± 2.17 (74.8–82.4)**	**27.0 ± 26.65 (78.4–67.7)**	76.0 ± 2.26 (72.0**–**80.1)	0.1 ± 1.48 (97.8**–**2.4)	10.8 ± 5.40 (98.9**–**20.3)	42.2 ± 5.57 (31.4**–**54.7)
R4	66.4 ± 2.70 (62.5**–**69.9)	98.5 ± 8.54 (82.2**–**16.2)	93.9 ± 4.29 (88.3**–**99.0)	37.6 ± 7.80 (17.6**–**48.7)	**87.8 ± 9.75 (60.9–99.2)**	**57.9 ± 11.15 (48.7–95.3)**	**12.9 ± 7.19 (95.1–23.5)**	**26.1 ± 4.25 (22.7–29.6)**
R5	81.5 ± 4.40 (72.7**–**90.4)	32.0 ± 9.50 (7.8**–**48.9)	17.5 ± 3.92 (10.3**–**24.8)	66.3 ± 2.47 (62.0**–**70.7)	NA	NA	NA	NA

Values in each cell represent the posterior mean ± SD (top) and the start and end values of the 95% HPD interval (bottom in parentheses). Both are reported as a percentage of standardized cycle time. Bolded cells indicate that there is no significant difference in timing between behaviors, thereby supporting the hypothesis.

There is no significant difference in the timing of minimum total tongue length between behaviors. During both behaviors, it occurs just prior to maximum gape (Fig. 5B andTable 3). Likewise, for R3, when minimum length occurs, there is also no statistical difference, despite on average occurring during FO and closing for chewing and drinking, respectively. This is because the timing of minimum R3 length during drinking has a lot of variation and a wide HPD interval (see Fig. 3 and Table 3). For all other regions (i.e., R1, R2, R4, and R5), there is a significant difference in the timing of minimum length between behaviors (Table 3). For chewing, minimum R1 length occurs during FO and sometimes during SO or at maximum gape, whereas for drinking, it occurs during closing or at maximum gape (Fig. 5B). R2 minimum length occurs around the SO–FO transition during chewing, whereas during drinking it occurs near maximum gape (Fig. 5B). For R4 it occurs just prior to maximum gape for chewing, whereas for drinking the mean occurs just prior to minimum gape (Fig. 5B). Finally, for R5 minimum length occurs during closing with a mean at the FC–SC transition for chewing whereas for drinking the mean is during O1 (Fig. 5B).

#### Width

**Fig. 6:**
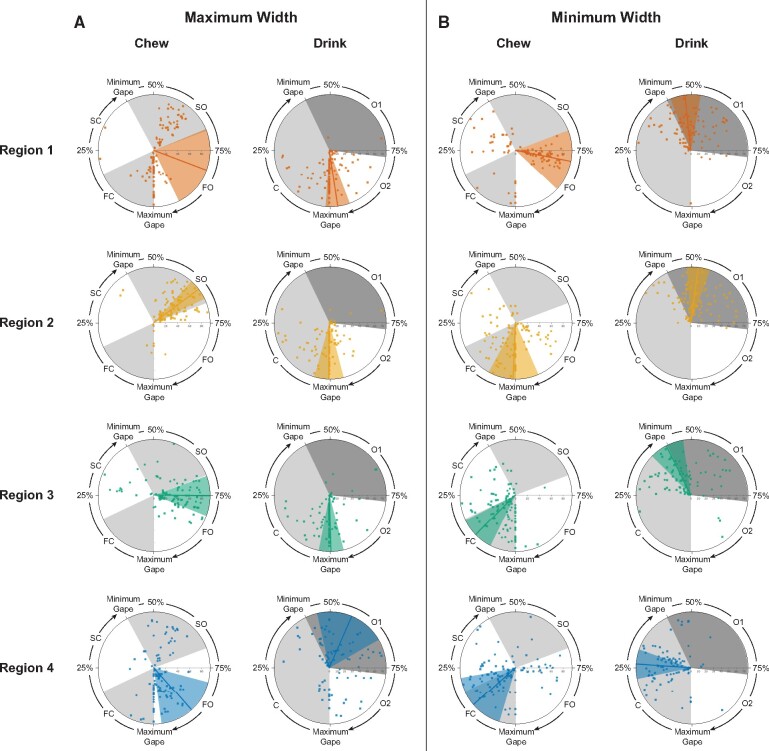
The timing of maximum and minimum width deformations relative to the gape cycle and mean phase durations were not always similar between behaviors. In each plot, the timing of maximum (left) or minimum (right) width deformation for each tongue region is expressed as a percent of total cycle duration and shown relative to wedges representing relative mean phase durations (alternating gray and white) during chewing and drinking (see Supplementary Figure S1). Lines indicate mean values and wedges show the corresponding variance. Individual 20 is indicated by circles and Individual 21 by squares. The location on the radius at 75% of the gape cycle indicates cycle number in the sequence with more centrifugal points corresponding to cycles later in the sequence.

We also expected the timing of maximum and minimum normalized width to occur at similar times during the gape cycle during chewing and drinking. This is only the case for maximum width for R1 and R4 as indicated by the overlapping chewing and drinking HPD intervals for each region (Table 3 and Fig. 6A). However, variability within R1 and R4, particularly during chewing may be driving this outcome. Specifically, during chewing, the timing of R1 maximum width is a bimodally distributed in both individuals (Fig. 6A). In some cycles, it reaches its maximum during SO, whereas in others it occurs around maximum gape, with a mean during FO. During drinking, maximum width occurs during O2 and closing, with a mean at maximum gape. For R4, there is a similar bimodal distribution of maximum width, but the mean is late during FO for chewing, whereas drinking maximum R4 width occurs throughout jaw opening, with a mean almost midway through O1. R2 and R3 each have nonoverlapping HPD intervals (Table 3). Maximum width occurs at the end of SO for R2 and after the start of FO for R3 during chewing, but for drinking, both regions reach maximum width around maximum gape (Fig. 6A).

For the timing of minimum width, HPD intervals for chewing and drinking only overlap for R4, with the mean of both occurring during jaw closing (Table 3 and Fig. 6B). In contrast, the width of R1 reaches its minimum on average during FO for chewing, but with a large spread throughout most of the cycle, whereas for drinking it clusters at the end of closing to O1, with a mean at the beginning of O1, just after minimum gape (Fig. 6B). For R2, the average minimum width occurs near maximum gape for chewing, with a large number of cycles in which it occurs during FO or after maximum gape during FC and SC. During drinking, R2 minimum width primarily occurs during O1 (Fig. 6B). Finally, the mean minimum width of R3 during chewing occurs during FC and at minimum gape for drinking (Fig. 6B).

### Relationship between regional length and widths—a test of the muscular hydrostat model

**Fig. 7:**
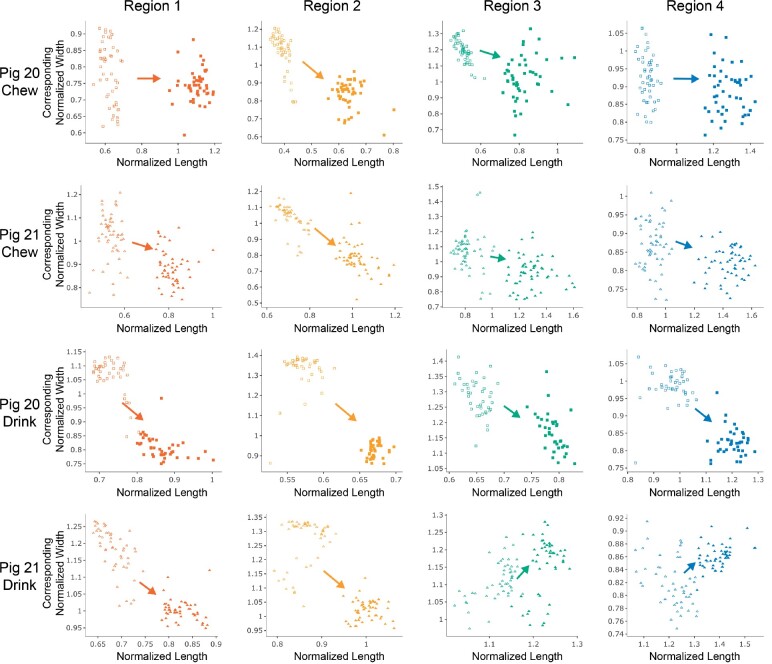
Regional normalized tongue length versus the corresponding width, demonstrating regional variability in the trade-off between tongue length and width. For each region, maximum AP versus corresponding ML deformations are plotted as one cloud (closed markers) and minimum AP versus corresponding ML deformations are plotted as another cloud (open markers). In accordance with the muscular hydrostat model, longer tongue widths would be expected to occur with narrow widths and shorter tongue lengths with higher widths. This would result in a point cloud in the upper left (short length and wide width) and lower right (long length and narrow width) of each panel. This trend, represented by the arrow, is generally observed here, with some variation. As we do not have dorsoventral tongue height, we would expect variation in the magnitude of the trade-off between AP and ML deformations, as observed here.

Regional differences in the relationship between tongue length and width are observed but are still consistent with the expectation that maximum length is associated with narrow widths and minimum length is associated with larger widths (Fig. 7). These patterns are more apparent in Pig 20 during drinking than during chewing, with R1 and R4 not showing the expected relationship at all. For Pig 21, this pattern is observed for all regions for chewing, but during drinking, R3 and R4 show the opposite relationship.

## Discussion

### Tongue deformations do not amplify tongue protraction and retraction during chewing and drinking

During chewing and drinking, the tongue protracts and retracts to position and move the bolus (Olson et al. 2021), and it is possible that AP lengthening and shortening contribute to these AP positional changes. At the level of the gape cycle, the tongue undergoes larger total and regional AP deformations and regional ML deformations during chewing than during drinking. These results mirror our previous finding that AP positional changes of the tongue, that is, tongue protraction and retraction, are greater during chewing (Olson et al. 2021), suggesting a mechanical link between AP movement and AP deformation, coupled with the concomitant changes in ML deformation due to the hydrostatic properties of the tongue. However, unlike the maximum and minimum length, which is reasonably similar between the 2 behaviors, the values of maximum and minimum widths are generally higher for drinking (see Table 2). This reflects the fact that the tongue is broader during drinking than chewing, but with a smaller amount of deformation throughout the cycle.

However, for both behaviors total tongue length is relatively conserved, suggesting that the relationship between tongue deformations and its AP positioning are not as tightly linked as hypothesized. Our results demonstrate that regional deformations do not contribute to major changes in total tongue length, due to their offset in timing, and thus are not an important mechanism to amplify tongue protraction. Moreover, compared to chewing, ΔL_cycle_ during drinking is lower even though the tongue stays in a more protracted state throughout the cycle. This may reflect that positional changes during drinking are also reduced (Olson et al. 2021). Considering the size of the anterior two-third of the pig tongue (∼114 mm long in Pig 20 and ~87 mm in Pig 21) and the distance between markers (here, 105.5 mm in Pig 20 and 72.5 mm in Pig 21), the total deformation of 4.9 mm (Individual 20) and 8.8 mm (Individual 21) that occurs during these behaviors is small. Our results are in agreeance with Liu et al. (2009) in which total length (and width) dimensional changes are typically less than ∼6 mm.

Despite small total tongue AP deformation during chewing and drinking, more substantial regional changes occur. R3–R5 undergo greater relative regional lengthening than do R1 and R2 (see Fig. 3). Moreover, larger deformations generally occur during chewing for these regions, owing to higher maximum and lower minimum normalized lengths. In fact, maximum normalized lengths for R1 and R2 are ∼1.0, indicating that these regions of the tongue rarely increase beyond resting length and that deformation largely occurs in a shortened state. In contrast, maximum normalized lengths for R3–R5 are >1.0. This increased elongation from R3–R5 offsets the shortened states of R1 and R2 to contribute to only slight increases in total tongue length during chewing and a relatively constant tongue length during drinking. During chewing, more so than during drinking, the timing of regional lengthening and shortening may also be important for regulating total tongue length during the cycle. The anterior regions tend to lengthen then shorten earlier in the gape cycle than the posterior regions, thereby further maintaining a relatively constant total length.

Previously, we showed that during chewing, maximum protraction of the anterior and posterior tongue markers occurs during FO, with the anterior marker on average slightly preceding the posterior marker (Olson et al. 2021). When compared to our data here, this timing is most clearly associated with the maximum lengthening of R4 and R5. However, the relationship between the timing of minimum tongue AP position (i.e., minimum protraction or maximum retraction) and minimum lengthening of R4 and R5 is less clear. This not only supports our conclusion above that the magnitude of positional changes of the tongue does not have to be temporally associated with similar magnitudes of deformational changes along the same axis, but that this relationship may also be variable with respect to polarity (i.e., lengthening versus shortening relative to protraction versus retraction).

This offset in the timing of regional AP deformations is also interesting given previous characterizations of tongue deformations by Thexton (1984). In some species, the tongue undergoes peristaltic deformations in the elevation of the ventral part of the tongue that is temporally offset from anterior to posterior to reposition the bolus. In other species, the anterior, middle, and posterior parts of the tongue contract and elongate out of phase with each other to alter overall tongue length. Our data are congruent with some aspects of this second mechanism barring the associated changes in tongue length, but we also cannot fully rule out the AP dorsoventral “peristalsis” without examining dorsoventral movements of the regional tongue markers. Regional tongue peristalsis will be examined in a future study by examining these movements.

### Chewing and drinking differ in the timing of tongue deformations relative to the gape cycle

We expected the timing of maximum and minimum AP and ML deformations relative to the gape cycle to be similar between behaviors, reflecting a general coordination between the tongue and jaw that facilitates function while also protecting the tongue from damage between the teeth as the jaw closes. Contrary to this hypothesis, we observed significant regional differences between chewing and drinking in the timing of maximum and minimum length for R1, R4, and R5 and for minimum length for R2 (see Fig. 5). We also observed differences between the behaviors in the timing of R2 and R3 maximum width and of R1–R3 minimum width (see Fig. 6). These results suggest regional functional differences between behaviors. For example, widening the middle of the tongue (R2 and R3) during jaw opening (see Fig. 6) may be associated with food positioning on the occlusal surfaces whereas a widening of this region during the opening phase of drinking may simply reflect sealing off the space between the occlusal surface and cheek to create a smaller, more midline, space for fluid transport. Nevertheless, the similarity between behaviors in when total tongue maximum and minimum AP deformation occurs (coupled with minimal changes in total tongue length), around minimum and maximum gape, respectively, suggests that the timing of tongue length changes may be conserved between behaviors, even when differences in the coordination between jaw and tongue movements (e.g., protraction–retraction) are observed between these same behaviors (see Olson et al. 2021).

Using sonomicrometry, Liu et al. (2009) previously found that maximum tongue length during chewing occurs midway through jaw closing, whereas here we found it occurring just after minimum gape, which occurs during their occlusal phase. Minimum tongue length is also later in our study, occurring just prior to maximum gape as opposed to the end of the occlusal phase or early opening. For drinking, Liu et al. (2009) found that maximum length occurs just after maximum gape and minimum length occurs early during jaw opening, likely corresponding to our O1 phase. Our results again show a delayed occurrence for both of these variables, with maximum length occurring toward the end of jaw closing closer to minimum gape, and minimum length occurring during the latter part of O2. As total tongue length is relatively constant in both behaviors, these differences in timing may not be functionally important. Rather, some of these differences likely reflect differences in experimental design between the 2 studies that would impact total length measurements, discussed below.

Sonomicrometry data reflect the absolute distance between crystals, not accounting for shape changes, especially changes in curvature, whereas we intentionally tried to capture these changes by summing the lengths of each region. This approach accounts for off-axis length changes to understand how regional deformations contribute to deformation of the overall structure. For some off-axis deformations, this could lead to marked differences in total lengths measured between the 2 methods. For example, arching of the tongue due to depression of the tip and elevation of the mid-region would be expressed as tongue shortening with sonomicrometry when, in fact, the tongue may be conserving its total length, or even lengthening, which would be captured by our approach. This is geometrically analogous to measuring chord length and arc length, respectively. More relevant to our dataset, however, is the apparent in- and out-of-plane twisting that occurs that would impact regional lengths (see Supplementary Videos S1 and S2). Interestingly, however, total tongue AP deformation is comparable in both studies and neither behavior resulted in pronounced tongue lengthening or shortening, further confirming our results that the timing, magnitude, and polarity of regional deformations contribute to maintaining tongue length within a narrow range.

The estimates of tongue width in both studies may be less subject to differences in experimental approaches given that this measurement is regionally defined here. Indeed, we found greater accordance between the 2 studies in regional tongue widths when we account for bead location. For the anterior tongue, corresponding to our R1, maximum width on average occurs midway through opening during chewing, which we more specifically identified as occurring during the FO phase. For drinking, maximum width of the anterior tongue occurs in both studies around maximum gape and minimum width occurs around minimum gape, specifically during the occlusal phase (Liu et al. 2009) and early O1 (this study). Given the lower kinematic resolution associated with skin markers to capture jaw movements in the earlier study, these are temporally reasonably coincident occurrences of anterior tongue width. The primary difference between the 2 studies for the anterior tongue was in the timing of minimum width during chewing. Liu et al. (2009) found that the anterior tongue is narrowest during chewing during the occlusal phase, whereas we observed this to occur during FO.

For posterior tongue width, represented here most closely by the widths of R3 or R4, there were also a number of similarities but some of these were specific to either R3 or R4 and not both, reflecting our finer scale regional characterization of ML deformations. During chewing, maximum width consistently occurs during jaw opening in both studies, whereas for drinking its occurrence during early jaw opening in Liu et al. (2009) was only similar to the timing associated with R4 (see Fig. 6A). In contrast, the timing of R3, and not R4, minimum width during drinking was similar between the 2 studies, occurring in both around minimum gape (see Fig. 6B). The one consistent difference between the 2 studies was minimum width during chewing. Here, R3 and R4 regions were narrowest during the early part of FC (see Fig. 6B), whereas Liu et al. (2009) observed minimum posterior width during late jaw closing, closer to the start of the occlusal phase.

In addition to the factors discussed above that may contribute to differences in total length calculations, any of the length and width differences between these datasets may reflect actual differences in timing. This raises questions about individual and cycle-to-cycle variation in jaw–tongue coordination. Indeed, for some differences noted above, a closer examination of the cycle-to-cycle variation observed here is not captured well by the mean. For example, maximum R1 width during chewing is bimodally distributed here between SO and around maximum gape and has a large variance, but the mean occurs during FO (see Fig. 6A), as in Liu et al. (2009). For R1 minimum width during chewing, there is also a large variance, and while most of the datapoints and the mean are within FO, there is a cluster of datapoints during SC (see Fig. 6B), which is what Liu et al. (2009) report. In an earlier study, Liu et al. (2007) specifically note that width changes are more variable with respect to jaw movements. This further emphasizes the potential impact of regional differences in variability as well as sampling differences on these comparisons.

### Regional AP and ML deformations reflect underlying anatomy and only partially support the muscular hydrostat model

**Fig. 8:**
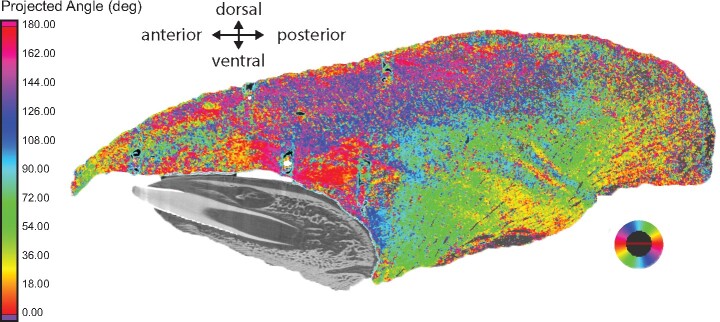
Mid-sagittal iodine-enhanced CT image of Individual 21, showing the muscle fiber directions of the tongue. This demonstrates the higher proportion of vertically and transversely oriented fibers in the posterior portion of the tongue, corresponding to the greater length changes observed in this area. Colors demonstrate the projected fiber angle relative to the mid-sagittal plane. Vertically orientated fibers running dorsoventrally through the tongue are colored blue and green, longitudinal fibers running anteroposteriorly are shown in red, and the highly interdigitated purple and pink areas have a high proportion of transversely oriented muscle fibers.

One of the main results of this study is that regional deformations differ, and that posterior tongue regions may undergo greater AP deformations beyond resting length than anterior regions. There may be an anatomical basis for these regional differences. In muscular hydrostats, increases in tongue length are driven by contraction of the vertically and transversely oriented fibers (Kier and Smith 1985; Smith and Kier 1989). Accordingly, the regions of the tongue where lengthening most occurs appear to have higher proportions of vertically and transversely oriented fibers (Fig. 8). Interestingly, this also suggests that there may be anatomical constraints for tongue lengthening in the anterior portion of the tongue, even though it is arguably the most freely mobile region. This portion of the tongue has a higher ratio of longitudinally oriented fibers and an increasing contribution of vertical fibers more posteriorly (Fig. 8). As tongue lengthening requires contraction of vertical and/or transverse fibers to reduce the cross-sectional area, this higher proportion of longitudinal fibers anteriorly poses a potential anatomical constraint for lengthening this region. Further, muscles act synergistically such that tongue protraction may include contributions from multiple intrinsic and extrinsic muscles during a single deformation (Napadow et al. 2002; Gilbert et al. 2007). There may also be passive stretch mechanisms from the contraction of extrinsic muscles that cause regional deformations within the tongue, as has been demonstrated by Napadow et al. (1999a). Depending on the timing of these contractions, there may be a compensatory effect on adjacent regions due to orthogonal expansion.

**Fig. 9:**
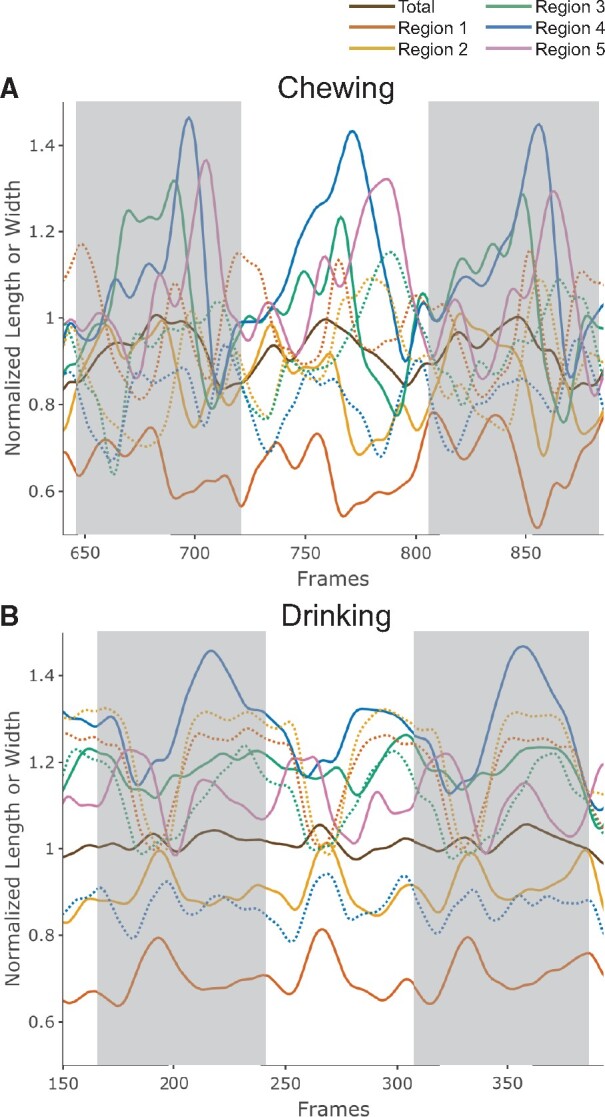
Normalized length and width of each region for 3 consecutive cycles of (**A**) chewing and (**B**) drinking for Individual 21. Solid and dashed lines are normalized lengths and widths, respectively, and demonstrate that there is not a clear trade-off in the timing of regional length and width deformations. Alternating grey and white boxes indicate gape cycles.

Although the muscular hydrostat model does not take into consideration the timing of extrinsic muscle contractions, it does predict synchronicity in the timing of orthogonal deformations. In other words, changes in one dimension must be compensated for by simultaneous deformation in others to maintain constant volume (Kier and Smith 1985; Smith and Kier 1989). As the timing of maximum and minimum regional deformations relative to the gape cycle only capture a single value, it is best to look at the trade-offs of length and width over time. During chewing, there are no whole-tongue patterns for the timing of length and width deformations (Fig. 9). This is counter to what would be expected from a muscular hydrostat. Instead, most length and width regions appear to increase in dimension throughout the middle parts of the cycle (Fig. 9A). The main exception to this is the width of R1, which has one peak occurring with the others, and a separate peak when most regions are at a lower normalized value. During drinking, a similar pattern is observed (Fig. 9B). However, the length of R1 and R2 and the width of R4 are shifted such that their maximal deformations are occurring when most regions are at their minimal deformations. Altogether, this suggests that changes in dorsoventral thickness also are important for characterizing hydrostatic deformations of the tongue.

Consistent with our hypothesis and as might be expected based on the AP deformations and the muscular hydrostat model, chewing is characterized by greater total ML deformations in all regions (Table 2). Although this is not directly evident from the datasets used for statistical analysis because of the offset in timing of our variables, when maximum and minimum lengths and their corresponding widths are compared (Fig. 7), we see that longer tongue lengths do not always correspond to narrower tongue widths. Even though we were not able to capture changes in the dorsoventral dimension (i.e., tongue height), there may still be a trade-off in length and width. The expected pattern is strongest for R1 and R2, whereas R3 and R4 show the opposite pattern for Pig 21 while drinking (Fig. 7). This suggests that there is regional biomechanical variability (e.g., differences in deformations and timings) throughout the structure and those muscular hydrostat properties are likely preserved at the whole-structure level. This is not surprising given that Liu et al. (2007, 2009) have shown regional volume changes in the tongue and that displacement of the tongue base posteriorly is not achieved by ML shortening of intrinsic tongue muscles during swallowing (Orsbon et al. 2020), which would be expected if the whole tongue functioned as a single muscular hydrostat.

The anatomical complexity of the tongue undoubtedly contributes to this biomechanical complexity. Napadow et al. (1999a) demonstrated different strain patterns throughout the tongue during different oral behaviors using diffusion-weighted MRI. Dorsoventral strain was demonstrated throughout bolus accommodation during human swallowing. This is attributed to contraction of vertically oriented fibers and led to expansion in both the AP and ML planes (Napadow et al. 1999a; Gilbert et al. 2007). Therefore, evaluation of dorsoventral regional changes would additionally benefit understanding of whether and, if so how, the tongue conforms to the expectation of a muscular hydrostat on a regional level.

### Limitations of the study and future directions

This study provides the most comprehensive data to date on regional tongue deformations during mammalian chewing and drinking, made possible by modifying methods for quantifying 3D movements of skeletal structures. However, the approach used here still presents challenges for interpretation. Because the tongue is not a rigid structure, marker implantation sites could not be systematically replicated between individuals, introducing some variability in the baseline/rest configuration of the tongue markers. In addition, markers potentially shifted before scarring into place, and this shift may be different between the 2 individuals. The impact of this potential difference could explain the individual difference in the contribution of regional AP contributions to total tongue length for R1 (15.62% versus 25.44%) and R4 (27.67% versus 17.12%) (Supplementary Table S1). Finally, the amount of the tongue captured by marker placement may differ, and therefore the regions may represent slightly different aspects of intrinsic tongue anatomy. Combined, these effects likely account for some of these differences observed between the 2 individuals. In spite of these differences, the individuals are generally comparable in the patterns of deformation observed (Figs. 3 and 4), with differences in absolute magnitude likely attributable to marker placement and/or individual variation. Future work aligning marker placement with the muscle insertions and fascicle orientations will enable a more specific anatomical context for interpreting these regional deformations.

The other major limitation is that the distance (i.e., lengths and widths) between markers reflects absolute linear distance between consecutive markers. Although this makes a solid approximation of length and width changes, it does not account for the other potential deformations that can also influence the distance between markers. For example, a decrease in distance is likely from shortening, but may also be influenced by bending, which may decrease the linear distance without decreasing actual muscle length. Specifically, minimum widths captured in the present dataset may include deformations other than shortening, such as ML bending or “rolling,” in which the distance between markers decreases but the actual tongue surface width is constant or may even increase. Our interpretations of the data cannot currently account for these potential confounding factors. However, this study does provide new critical insights into the regional contributions to tongue deformations and serves as the basis for future work on multidimensional shape changes during oral behaviors.

## Supplementary Material

obab012_Supplementary_Data
