# Supplementary material for: Regional Tongue Deformations During Chewing and Drinking in the Pig
Source: Integr Org Biol. 2021 Apr 22;3(1):obab012. doi: 10.1093/iob/obab012 (PMC8601049; doi:10.1093/iob/obab012)
Supplement: obab012_Supplementary_Data [file obab012_supplementary_data.zip › Supplemental_IOB-2021-006.docx]

**Supplemental Table 1.** Contribution of each region to total tongue length and width

|  | **% of total tongue length^a^** | | | | | **% of the widest right-left marker pair** | | | |
| --- | --- | --- | --- | --- | --- | --- | --- | --- | --- |
| **Individual ID** | **R1** | **R2** | **R3** | **R4** | **R5** | **R1** | **R2** | **R3** | **R4** |
| **Pig 20** | 15.62% | 17.75% | 18.24% | 27.67% | 20.71% | 100% | 62.52% | 55.05% | 64.76% |
| **Pig 21** | 25.44% | 18.51% | 18.97% | 17.12% | 19.97% | 62.79% | 80.98% | 87.46% | 100% |

^a^ Total tongue length is the sum of the length between the anterior midline marker and posterior midline marker of each region.

**Supplemental Figure 1**


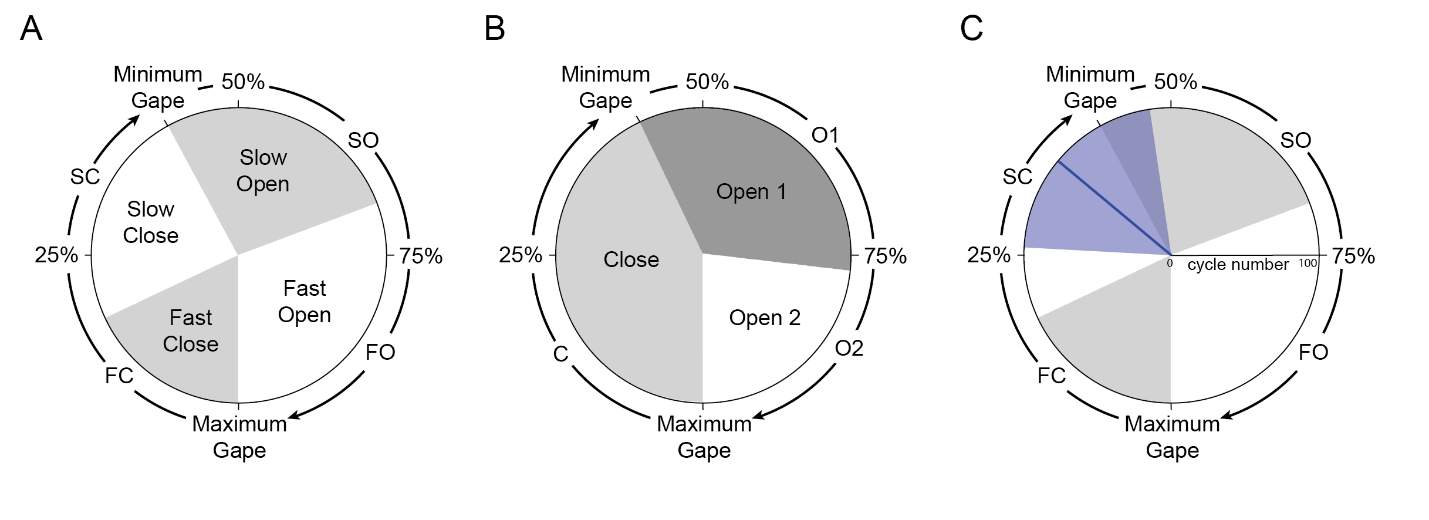


**Supplemental Figure 1.** Schematic representation of mean cycle phase duration for (A) chewing and (B) drinking, using values from Olson et al., 2021 on the same dataset. Panel (C) indicates the radius at 75% separates data points by cycle number, so individual datapoints are visible. Additionally, the solid blue radius line indicates an example of a circular mean occurring during slow close with the blue transparent wedge indicating the corresponding variance.

**Supplemental Video 1.** XROMM animation of Pig 20 chewing a piece of apple.

**Supplemental Video 2.** XROMM animation of Pig 20 drinking.
